# The anterior scleral thickness in eyes with primary open-angle glaucoma

**DOI:** 10.1007/s00417-021-05523-3

**Published:** 2022-01-24

**Authors:** Xiaoqin Yan, Mu Li, Zhiqi Chen, Xiongwu Zhou

**Affiliations:** 1 Department of Ophthalmology, Tongji Hospital, Tongji Medical College, Huazhong University of Science and Technology, Wuhan, 430030 China; 2 Department of Ophthalmology, Union Hospital, Tongji Medical College, Huazhong University of Science and Technology, Wuhan, 430022 China

**Keywords:** Primary open-angle glaucoma, Anterior scleral thickness, Schlemm’s canal, Trabecular meshwork, Swept-source optical coherence tomography

## Abstract

**Purpose:**

To investigate the anterior scleral thickness (AST) and its associations with Schlemm’s canal (SC) area, trabecular meshwork (TM) thickness and length, and scleral spur (SS) length in healthy and primary open-angle glaucoma (POAG) groups.

**Methods:**

Thirty-five eyes of 35 healthy subjects and 23 eyes of 23 patients with POAG were included. The AST, SC area, TM thickness and length, and SS length were measured using swept-source optical coherence tomography. AST was measured at 0 mm (AST0), 1 mm (AST1), 2 mm (AST2), and 3 mm (AST3) from SS. Associations between AST and SC area, TM thickness and length, and SS length were also estimated.

**Results:**

AST0 (728.84 ± 99.33 vs. 657.39 ± 67.02 μm, *p* < 0.001), AST1 (537.79 ± 79.55 vs. 506.83 ± 57.37 μm, *p* = 0.038), AST3 (571.09 ± 79.15 vs. 532.13 ± 59.84 μm, *p* = 0.009), SC area (6304.26 ± 1238.72 vs. 4755.64 ± 1122.71 μm^2^, *p* < 0.001), TM thickness (107.21 ± 31.26 vs. 94.51 ± 24.18 μm, *p* = 0.035), TM length (736.20 ± 141.85 vs. 656.43 ± 127.03 μm, *p* = 0.004), and SS length (219.89 ± 50.29 vs. 174.54 ± 35.58 μm, *p* < 0.001) were significantly greater in healthy group than in POAG group. In addition, SC area, TM thickness, and SS length were significantly and positively associated with AST0 in the healthy group, whereas no similar associations were observed in the POAG group.

**Conclusions:**

Compared with the healthy group, AST was significantly thinner in the POAG group, which also had smaller SC and TM dimensions. Moreover, the SC area, TM thickness, and SS length were significantly and positively associated with AST in the healthy group. Thus, AST might play an important role in maintaining TM and SC morphology and further in the pathogenesis of POAG.


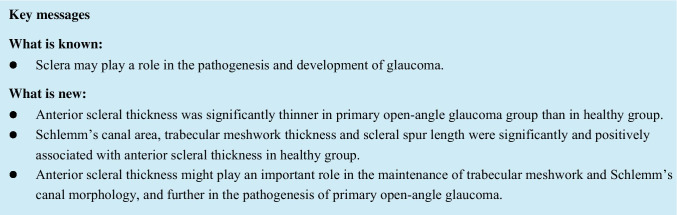


## Introduction

The sclera forms the majority of the outer layer of the eyeball, acting as a support and anchor for the more delicate intraocular structures and tissues [1]. A recent study indicated that the sclera may play a major role in the pathogenesis of some ocular diseases [2, 3]. In terms of glaucoma, for example, previous studies have reported that intraocular pressure (IOP)-induced scleral deformations could be transmitted to the lamina cribrosa and optic nerve head tissues, leading to axonal damage of retinal ganglion cells [4, 5]. In addition, the biomechanical response of the lamina cribrosa and optic nerve head tissues to IOP could be determined by the posterior sclera thickness (PST) [6–8]. In the eyes with thinner PST, the biomechanical properties of the lamina cribrosa and optic nerve head tissues are suggested to be abnormal, resulting in increased susceptibility to glaucomatous retinal ganglion cell damage [9, 10].

The elevated IOP in primary open-angle glaucoma (POAG) is caused by an increase in the aqueous humor outflow resistance, predominantly in the trabecular meshwork (TM) and Schlemm’s canal (SC) [11, 12]. In addition, previous studies have reported that the abnormality in TM and SC dimensions (compressed TM and collapsed SC) might contribute to increased aqueous humor outflow resistance in eyes with POAG [13, 14]. Another previous study of healthy subjects also indicated that the expansion of the TM and SC could lead to a reduction in IOP [15]. Thus, the changes in TM and SC dimensions (compressed TM and collapsed SC) might play a key role in the increase in aqueous humor outflow resistance in patients with POAG.

The sclera plays a role in determining the biomechanical environment of ocular tissues, including the cornea [5, 16]. The TM and SC are situated in the limbus, which is located right between the cornea and anterior sclera, and previous studies have suggested that the limbus contains scleral elements [17, 18]. In addition, the scleral spur (SS), which is part of the anterior sclera, has also been reported to be an important factor for maintaining TM and SC morphology [19, 20]. Thus, changes in the anterior scleral thickness (AST) may also influence the morphology of the TM and SC. However, although the PST was proven to be thinner in eyes with POAG [9], it remains unclear whether the AST is linearly correlated with the PST. Accordingly, in this study, we aimed to measure the AST and to investigate its correlations with the dimensions of the TM and SC in healthy and POAG groups.

## Materials and methods

This study was approved by the ethics committee of Tongji Hospital, Huazhong University of Science and Technology, and adhered to the tenets of the Declaration of Helsinki. All subjects provided written informed consent prior to study participation.

### Study subjects

Thirty-five healthy eyes of 35 healthy subjects and 23 POAG eyes of 23 patients with POAG were included in this study. The recruited subjects received refractive error (RE) examination (RT-2100, NIDEK CO. LTD., Gamagori, Japan), central corneal thickness (CCT) measurement (corneal map, swept-source optical coherence tomography (SS-OCT), CASIA SS-1000, Tomey Corp., Nagoya, Japan), axial length (AL) measurement (IOL-Master, Carl Zeiss Meditec, Dublin, CA, USA), IOP measurement, gonioscopy, slit-lamp examination, fundus photography, retinal nerve fiber layer (RNFL) thickness measurement (spectral domain (SD)-OCT, Heidelberg Engineering GmbH, Heidelberg, Germany), and standard automated perimetry examination (Humphrey Field Analyzer II, Carl Zeiss Meditec, Dublin, CA, USA). Subjects were included in the POAG group if (1) at least 18 years old and (2) meet the diagnostic criteria of POAG: open anterior chamber angle on gonioscopy; glaucomatous optic neuropathy: cup disc ratio of > 0.7 or inter-eye asymmetric optic disc ratio of > 0.2 and/or glaucomatous notching; compatible visual field loss. Glaucomatous visual field loss was defined by the Humphrey visual field results (Humphrey Field Analyzer II, Carl Zeiss Meditec, Dublin, CA, USA): three contiguous points within the same hemifield on the pattern deviation probability at p < 5%, with at least one point at *p* < 1%, and the glaucomatous hemifield test result beyond normal limits. Cutoff for low visual field test reliability was 20% fixation losses, 33% false positives, and 33% false negatives [21, 22]. Patients with a history of ocular surgery or systemic disease (e.g., hypertension or diabetes mellitus) were excluded from the study. All patients with POAG were being treated with anti-glaucoma medications. Healthy subjects were defined as (1) at least 18 years old, (2) IOP of ≤ 21 mmHg with no history of IOP elevation, (3) normal fundus, (4) no visible RNFL defects, (5) normal visual field, and (6) open anterior chamber angle. Subjects with a family history of glaucoma, prior ocular surgery, or systemic disease were excluded from the study. One eye was randomly selected from each subject for SS-OCT examinations (CASIA SS-1000; Tomey Corp., Nagoya, Japan).

### SS-OCT imaging acquisition and processing

The recruited subjects were imaged with the high-density (HD) scan of SS-OCT. The participants were instructed to open the eye wide during an examination, and the nasal and temporal limbi were recorded separately after adjusting the fixture to the corresponding areas. The scans were performed three times and the image with the best quality was chosen for analysis.

### Measurements of the TM, SC, SS, and AST

**Fig. 1:**
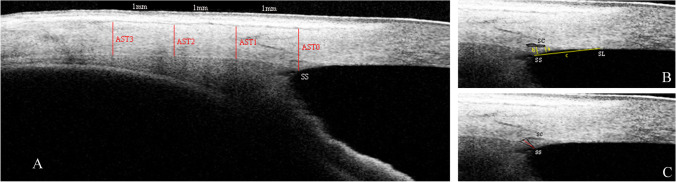
**A** The measurements of the anterior scleral thickness (AST, red line). **B** The measurements of TM thickness (yellow line a and b), TM length (yellow line c), and SC area (black curve). **C** The measurement of SS length (red line); SC, Schlemm’s canal; SS, scleral spur; SL, Schwalbe’s line

As described in our previous studies, SC, TM, and SS, SC were defined as a thin, black, and lucent space in the HD image, and the SC area was manually drawn freehand based on the outlines of SC [20]. The TM thickness was calculated as the average value of two measurements conducted at the halfway point and anterior endpoint of the inner wall of the SC. Each measurement of TM thickness was conducted perpendicular to the inner layer of the TM, beginning at the SC inner wall [14, 15]. The TM length was defined as the length from the hyperreflective point closest to the SS to the hyperreflective point closest to Schwalbe’s line [23, 24]. The SS length was defined as the length of the line bisecting the width of the SS at every point (the line started from the tip of the SS to the middle of the anterior and posterior points where the sclera curves out to form the spur) [20, 25]. The AST was defined as the distance from the episcleral blood vessels (a thin hyporeflective area in the anterior part of the sclera) to the posterior boundary of the sclera (the line separating the hyperreflective sclera from the hyporeflective ciliary muscle) [26] (Fig. 1). The AST was measured at 0 mm (AST0), 1 mm (AST1), 2 mm (AST2), and 3 mm (AST3) from the SS. All measurements were performed using ImageJ software (National Institutes of Health, Bethesda, MD, USA), and the measurements were masked to the study information.

### Statistical analysis

All analyses were conducted by R software version 3.4.3 (https://www.r-project.org). Data were presented as mean ± standard deviation (SD) where applicable. Differences in continuous variables between two groups were compared using independent-samples *t* tests, differences in categorical variables were compared using the chi-square test. Differences in parameters including nasal and temporal measurement results were compared using the general estimate equations (GEEs), which take into account the correlation between the measurements from nasal and temporal quadrants of one eye. Linear regression was used to determine the associations between SC area, TM thickness, TM length, and AST, and the associations between SS length and AST. Adjusted *β* coefficients for the associations between independent and dependent variables were assessed using GEEs. To evaluate the intraobserver variance, all the parameters were re-measured by the same experienced observer (ML) at a separate session. The intraobserver reproducibility was assessed by the intraclass correlation coefficient (ICC). All tests were two-tailed, and statistical significance was defined as a *p* value of < 0.05.

## Results

The demographic characteristics of the study subjects are shown in Table 1. There were no significant differences in terms of age, sex, CCT, AL, and RE between the healthy and POAG groups (all *p* > 0.05). The IOP was significantly lower in the healthy group than in the POAG group (15.39 ± 2.44 vs. 20.94 ± 5.54 mmHg, *p* < 0.001), whereas the SC area (6304.26 ± 1238.72 vs. 4755.64 ± 1122.71μm^2^, *p* < 0.001), TM thickness (107.21 ± 31.26 vs. 94.51 ± 24.18 μm, *p* = 0.035), TM length (736.20 ± 141.85 vs. 656.43 ± 127.03 μm, *p* = 0.004), and SS length (219.89 ± 50.29 vs. 174.54 ± 35.58 μm, *p* < 0.001) were significantly greater in the healthy group compared with the POAG group. The AST was significantly thicker in the healthy group than in the POAG group at most locations (AST0: 728.84 ± 99.33 vs. 657.39 ± 67.02 μm, *p* < 0.001; AST1: 537.79 ± 79.55 vs. 506.83 ± 57.37 μm, *p* = 0.038; AST3: 571.09 ± 79.15 vs. 532.13 ± 59.84 μm, *p* = 0.009), with the exception of AST2 (552.41 ± 71.78 vs. 526.26 ± 61.76 μm, *p* = 0.083). For the POAG group, the visual field index was 60.22 ± 31.38%, the mean deviation was − 14.38 ± 9.27 dB, and pattern standard deviation was 8.53 ± 4.76 dB.

**Table 1:** Characteristics of study subjects

	Healthy group	POAG group	*p*
Age (years)	37.3 ± 12.8	40.3 ± 12.8	0.382^a^
Sex (male/female)	16/19	15/8	0.184^b^
CCT (μm)	536.54 ± 25.32	533.09 ± 29.07	0.634^a^
AL (mm)	23.65 ± 0.83	23.27 ± 0.89	0.102^a^
RE (D)	− 0.68 ± 1.00	− 0.18 ± 1.37	0.122^a^
IOP (mmHg)	15.39 ± 2.44	20.94 ± 5.54	< 0.001^a^*
SC area (μm^2^)	6304.26 ± 1238.72	4755.64 ± 1122.71	< 0.001^c^*
TM thickness (μm)	107.21 ± 31.26	94.51 ± 24.18	0.035^c^*
TM length (μm)	736.20 ± 141.85	656.43 ± 127.03	0.004^c^*
SS length (μm)	219.89 ± 50.29	174.54 ± 35.58	< 0.001^c^*
AST0 (μm)	728.84 ± 99.33	657.39 ± 67.02	< 0.001^c^*
AST1 (μm)	537.79 ± 79.55	506.83 ± 57.37	0.038^c^*
AST2 (μm)	552.41 ± 71.78	526.26 ± 61.76	0.083^c^
AST3 (μm)	571.09 ± 79.15	532.13 ± 59.84	0.009^c^*

*CCT*, central corneal thickness; *AL*, axial length; *RE*, refractive error; *IOP*, intraocular pressure; *SC*, Schlemm’s canal; *TM*, trabecular meshwork; *SS*, scleral spur; *AST*, anterior scleral thickness

^a^Independent-samples *t* test, ^b^chi-square test, ^c^general estimate equations

^*^Significance of differences

### Comparisons of the CCT, SC area, TM length and thickness, SS length, and AST between POAG subgroups (anti-glaucoma treatment with and without prostaglandin analogs)

We divided the patients with POAG into the following two subgroups: those receiving anti-glaucoma treatment with prostaglandin analogs (PGAs) (*n* = 14) and those receiving anti-glaucoma treatment without PGAs (*n* = 9). Subsequently, we compared the CCT, SC area, TM length and thickness, SS length, and AST between these two subgroups. The results showed that all the ocular parameters were not significantly different between these two POAG subgroups (all *p* > 0.05) (Table 2).

**Table 2:** Comparisons of CCT, SC, TM, SS, and AST between POAG subgroups (anti-glaucoma treatment with and without PGAs)

POAG			
	Anti-glaucoma treatment with PGAs (*n* = 14)	Anti-glaucoma treatment without PGAs (*n* = 9)	
CCT (μm)	530.14 ± 30.30	537.67 ± 26.32	0.519^a^
SC area (μm^2^)	4568.27 ± 1067.54	4998.12 ± 1177.77	0.228^b^
TM thickness (μm)	98.50 ± 27.05	89.35 ± 19.44	0.218^b^
TM length (μm)	670.75 ± 126.63	634.17 ± 128.02	0.320^b^
SS length (μm)	174.82 ± 41.97	174.18 ± 26.37	0.956^b^
AST0 (μm)	669.71 ± 59.60	638.22 ± 74.89	0.190^b^
AST1 (μm)	512.11 ± 54.16	498.61 ± 62.73	0.520^b^
AST2 (μm)	530.82 ± 62.84	519.17 ± 61.14	0.601^b^
AST3 (μm)	532.21 ± 64.96	532.00 ± 52.71	0.991^b^

*PGAs*, prostaglandin analogs; *CCT*, central corneal thickness; *SC*, Schlemm’s canal; *TM*, trabecular meshwork; *SS*, scleral spur; *AST*, anterior scleral thickness

^a^Independent-samples *t* test, ^b^general estimate equations

### Associations between the SC area, TM thickness, TM length, and AST

In the healthy group, AST0 (*β* = 0.025, *p* = 0.016) was significantly associated with the SC area, and AST0 (*β* = 0.915, *p* = 0.006) and AST3 (*β* = 0.691, *p* = 0.025) was significantly associated with TM thickness. In contrast, in the POAG group, no significant associations were found between the SC area, TM thickness, TM length, and AST (Table 3).

**Table 3:** Associations between SC area, TM thickness, TM length, and AST

Healthy group								
	AST0		AST1		AST2		AST3	
SC area	*β*	*p*	*β*	*p*	*β*	*p*	*β*	*p*
	0.025	0.016*	0.016	0.064	0.011	0.137	0.012	0.231
TM thickness	*β*	*p*	*β*	*p*	*β*	*p*	*β*	*p*
	0.915	0.006*	0.401	0.152	0.316	0.132	0.691	0.025*
TM length	*β*	*p*	*β*	*p*	*β*	*p*	*β*	*p*
	0.064	0.424	0.046	0.423	0.051	0.213	0.065	0.213
POAG group								
	AST0		AST1		AST2		AST3	
SC area	*β*	*p*	*β*	*p*	*β*	*p*	*β*	*p*
	0.002	0.841	0.004	0.613	− 0.002	0.641	0.008	0.181
TM thickness	*β*	*p*	*β*	*p*	*β*	*p*	*β*	*p*
	0.311	0.284	− 0.272	0.316	0.116	0.734	-0.077	0.835
TM length	*β*	*p*	*β*	*p*	*β*	*p*	*β*	*p*
	0.106	0.121	0.078	0.090	− 0.035	0.483	− 0.106	0.104

*β/P* value: The influence factors as age, sex, axial length, central corneal thickness, and refractive error have been adjusted

^*^Significance of differences: general estimate equations

*SC*, Schlemm’s canal; *TM*, trabecular meshwork; *AST*, anterior scleral thickness

### Associations between SS length and AST

In the healthy group, AST0 (*β* = 0.52, *p* = 0.004) and AST3 (*β* = 0.34, *p* = 0.028) were significantly associated with SS length, whereas no significant association between the SS length and AST was observed in the POAG group (Table 4).

**Table 4:** Associations between SS length and AST

Healthy group								
	AST0		AST1		AST2		AST3	
SS length	*β*	*p*	*β*	*p*	*β*	*p*	*β*	*p*
	0.52	0.004*	0.22	0.151	0.20	0.066	0.34	0.028*
POAG group								
	AST0		AST1		AST2		AST3	
SS length	*β*	*p*	*β*	*p*	*β*	*p*	*β*	*p*
	0.47	0.147	0.31	0.205	0.16	0.618	0.16	0.570

*β/P* value: The influence factors as age, sex, axial length, central corneal thickness, and refractive error have been adjusted

^*^Significance of differences: general estimate equations

*SS*, Scleral spur; *AST*, anterior scleral thickness

### Reproducibility of the measurements of the SC, TM, SS, and AST parameters

The results shown in Table 5 indicate that the reproducibility of the measurements was good. The ICC values of the measurements ranged from 0.865 to 0.941.

**Table 5:** Reproducibility of the measurements of the SC, TM, SS, and AST parameters

		95% confidence intervals	
	ICC	Lower	Upper
SC area (μm^2^)	0.865	0.806	0.907
TM thickness (μm)	0.866	0.807	0.907
TM length (μm)	0.873	0.822	0.910
SS length (μm)	0.894	0.847	0.927
AST0 (μm)	0.941	0.916	0.959
AST1 (μm)	0.925	0.893	0.947
AST2 (μm)	0.918	0.884	0.943
AST3 (μm)	0.891	0.847	0.923

*SC*, Schlemm’s canal; *TM*, trabecular meshwork; *SS*, scleral spur; *AST*, anterior scleral thickness; *ICC*, intraclass correlation coefficient

## Discussion

In the present study, we compared the AST between healthy individuals and those with POAG: with the exception of AST2, the AST was significantly thicker in the healthy group than in the POAG group at all measurement locations (0 mm, 1 mm, and 3 mm from the SS). Moreover, we divided the POAG group into two subgroups: anti-glaucoma treatment with and without PGAs. The comparisons of the CCT, TM, SC, SS, and AST between these two subgroups revealed that none of the parameters significantly differed. Furthermore, in the healthy group, the TM thickness, SC area, and SS length were significantly associated with AST0; the TM thickness and SS length were significantly associated with AST3. In contrast, no such significant associations were observed in the POAG group.

The sclera is the major stress-bearing component of the eye and accounts for approximately 90% of the outer layer of the eyeball. Differences in scleral properties can affect the mechanical properties of the eye and its biomechanical response to IOP [4, 27]. Thus, scleral properties may be central to various aspects of glaucoma development and treatment [2]. Previous studies have already indicated that POAG is accompanied by alterations in the proteome of the sclera, which is associated with changes in scleral properties [28]. However, unlike other parameters, such as the CCT and PST, which have been previously reported to be powerful predictors of glaucoma development [9, 10, 29], little is known about the role of the AST in the pathogenesis of glaucoma.

Approximately 75 to 80% of aqueous humor secreted by the ciliary body flows through the conventional TM and SC pathways back into the circulatory system [30]. The major site of aqueous humor outflow resistance is in the juxtacanalicular tissue of the TM and the SC inner wall [11, 12]. The elevated IOP could result in the compression of TM and the collapse of SC, which would further increase the aqueous humor outflow resistance in return [31]. Conversely, the expansion of the TM and SC could lead to a reduction in IOP [15, 32]. Moreover, the morphology of the TM and SC are both abnormal (compressed TM and collapsed SC) in POAG eyes compared with healthy controls [13, 14]. Thus, the status of the TM and SC is important in the pathogenesis of POAG.

In this study, we found that AST0, AST1, and AST3 were significantly thinner in the POAG group than in the healthy group. A previous study reported that long-term (1 year) administration of PGAs could induce a decrease in AST in patients with POAG [33]. As the application of PGAs could act as a confounding factor when assessing changes in glaucomatous AST, we compared the CCT, TM, SC, SS, and AST between two POAG subgroups (anti-glaucoma treatment with and without PGAs). The results showed that none of the ocular parameters significantly differed between the two POAG subgroups (with and without PGAs), indicating that the administration of PGAs induced no changes in AST of our POAG patients, and the effect of PGAs on AST could be excluded in this present study. Accordingly, the decrease in AST of POAG patients observed in this study might not be secondary to the administration of PGAs, but rather be primary. POAG patients might have original thinner anterior sclera and healthy subject might have original thicker anterior sclera anatomically.

Using SS-OCT, Dhakal et al. previously measured the AST at different distances (0–5 mm) from the SS and found that AST0 was the thickest (temporal: 714 μm; nasal: 653 μm) among all the measured locations, with the thicknesses of AST1, AST2, AST3 being approximately 550 μm [26]. Their study results are highly consistent with our own. Using SD-OCT, similar results were observed by Woodman-Pieterse et al., who reported measurements of AST1, AST2, and AST3 of 512 μm, 504 μm, and 543 μm, respectively [34]. In terms of AST comparisons between healthy individuals and those with POAG, a previous study involving anterior segment (AS)-OCT revealed no significant difference in the AST between healthy eyes and those with POAG [35], which is inconsistent with the results of the present study. The underlying reason for this discrepancy might be related to differences in the measurement methods used and the definitions of AST. In the present study, we defined AST as the measured distance between the episcleral blood vessels and the interface between the sclera and ciliary body. However, the previous AS-OCT study [35] defined the AST as the measured distance between the first high reflective tissue signal of the episclera and the interface between the sclera and ciliary body. Thus, their AST measurements could have included episcleral tissues [17, 35]. The inclusion of episcleral tissues in the AST measurements could also explain why their AST values were significantly greater (AST2: 784 μm for the healthy group and 772 μm for the POAG group) than our own (AST2: 552 μm for the healthy group and 526 μm for the POAG group). Ultrasound biomicroscopy (UBM) studies have previously investigated the AST; however, UBM examination requires the use of an eyecup, which may affect the measurement of the original AST. Moreover, the resolution of UBM could be much lower than that of OCT, which could also lead to measurement differences [35, 36]. Thus, the results from previous UBM studies seem to be not comparable to our SS-OCT results.

We also measured SC and TM parameters in this study and found that the SC area, TM length, and TM thickness were significantly smaller in the POAG group than in the healthy group, which is consistent with the results of previous SC and TM-related studies. By postmortem eyes, Allingham et al. found that the SC area, SC perimeter, and SC length were significantly smaller in POAG eyes than in normal eyes, and the reduction in SC dimensions may have contributed to nearly half of the reduction in outflow facility observed in the POAG eyes [37]. With the development of newer observational methods, UBM and OCT have provided a means of evaluating SC in vivo. Using UBM, Yan et al. reported a smaller SC in eyes with POAG than in normal eyes [14]. Similar results were also observed in OCT studies, indicating a decrease in SC dimensions in POAG eyes [13, 20, 38]. In addition, the SC area could also be a clinical predictor of IOP reduction amount prior to the application of anti-glaucoma medications in patients with POAG [39]. In terms of the TM, previous studies have found that the TM might become compressed with an acute increase in IOP [31], and the TM expansion induced by Y27632 could increase the outflow facility [40]. Compared with normal eyes, Stegman et al. reported that TM is shorter in juvenile POAG eyes [41], and Yan et al. reported that TM is thinner in POAG eyes [14]. The results of both of those studies indicated the presence of morphological abnormalities in the TM of the eyes with POAG. Moreover, the TM differed in size according to ethnicity, and ethnicities with a higher prevalence of glaucoma were suggested to have a shorter TM. Thus, TM size may be a novel risk factor for POAG [42]. The length of the TM was found to be shorter in eyes affected by angle closure glaucoma than in eyes with POAG, and a shorter TM could be part of the pathophysiology driving angle closure [24, 43].

As mentioned above, the AST, TM thickness, TM length, SC area, and SS length were all significantly greater in the healthy group than in the POAG group in this study. The thicker anterior sclera in the healthy group might be better able to support the morphology of the TM and SC, maintaining the expansion of TM and the opening of SC to reduce the aqueous humor outflow resistance. Furthermore, the SS has long been assumed to be a supporting tissue for the TM and SC, as a shorter SS could not comprehensively support the TM and SC, resulting in the pathogenesis of POAG [20, 25, 44]. The SS is part of the anterior sclera, and AST might have an impact on SS length. Accordingly, we speculated that the thinner anterior sclera observed in the POAG group might have led to a shorter SS length, resulting in insufficient support for the patency of the TM and SC. The compressed TM and collapsed SC could further increase the aqueous humor outflow resistance, leading to the increase in IOP and pathogenesis of POAG [20, 25, 44, 45].

The thinner anterior sclera of patients with POAG might also have implications for drug delivery and surgical interventions. Drug delivery through the sclera provides another option for vitreous and retinal therapy, and this less invasive approach is safer than intravitreal injection [46, 47]. Moreover, transscleral drug delivery can also provide localized and sustained drug release [46]. The scleral permeability plays an important role in transscleral drug delivery. Scleral thickness has been reported to be inversely related to scleral permeability, with a thinner sclera accompanied by greater scleral permeability [47, 48]. Thus, for patients with POAG, whose anterior sclera was found to be thinner in this study, transscleral drug delivery might be more effective and efficient. In terms of surgical interventions, such as scleral buckling or strabismus surgeries, the sutures pass through the sclera lamella [48]. Considering the thinner anterior sclera in patients with POAG, the surgeon should pay more attention to the sutures by POAG eyes during such surgeries, and perhaps the sutures should be placed slightly more superficially than usual. Moreover, there is evidence suggesting that repeated intravitreal injections in the same quadrant could result in scleral thinning [49]. Thus, in patients with POAG, repeated intravitreal injections should probably be performed in different quadrants instead of being administered in the same quadrant. Another report of complication caused by intravitreal injection indicated that the thinner sclera might be the potential reason for the accidental intralenticular Ozurdex (dexamethasone implant) injection [50].

In this study, the association analysis revealed that the TM thickness, SC area, and SS length were positively associated with AST0 in the healthy group, indicating that the thicker anterior sclera might result in a thicker TM, larger SC, and longer SS, which is consistent with the aforementioned speculation that a thicker anterior sclera could better support the morphology of the TM, SC, and SS. However, in the POAG group, no such associations were observed. The underlying reason might be that the morphology of the TM and SC in the POAG group can also be affected by other pathophysiological factors, such as changes in the autonomic nervous system [15, 32, 51, 52], alterations in the TM and SC stiffness [53–56], and differences in the extracellular matrix of the TM and SC [55, 57]. Therefore, the morphology of the TM and SC in POAG might be determined by multiple factors and may not be solely explained by the AST.

This study has certain limitations. First, our sample size was relatively small. Expanding our sample size might lead to greater statistical power and more sensitive findings. Second, the patients with POAG were relatively young (40.3 ± 12.8 years old). Although previous studies have indicated that age does not significantly influence AST [58, 59], it remains unclear whether similar results would be observed in older groups. Third, all of the subjects in the study were Chinese. The previous study has reported ethnic differences in the AST. The AST of Caucasians was thinner than that of non-Caucasians [17]. Thus, it is unclear whether similar results would be observed in other ethnic groups.

In conclusion, AST0, AST1, and AST3 were significantly thinner in eyes with POAG than in healthy eyes. The SC area, TM thickness, and SS length were significantly and positively associated with AST0 in the healthy group. The thinner anterior sclera might have contributed to the smaller TM and SC dimensions in the POAG group. Thus, the AST might play an important role in maintaining SC and TM morphology and in the pathogenesis of POAG.

## Data Availability

The datasets generated and/or analyzed during the current study are available from the corresponding author on reasonable request.
